# Prevalence of single nucleotide polymorphism among 27 diverse alfalfa genotypes as assessed by transcriptome sequencing

**DOI:** 10.1186/1471-2164-13-568

**Published:** 2012-10-29

**Authors:** Xuehui Li, Ananta Acharya, Andrew D Farmer, John A Crow, Arvind K Bharti, Robin S Kramer, Yanling Wei, Yuanhong Han, Jiqing Gou, Gregory D May, Maria J Monteros, E Charles Brummer

**Affiliations:** 1 The Samuel Roberts Noble Foundation, 2510 Sam Noble Parkway, Ardmore, OK, 73401, USA; 2 Institute of Plant Breeding, Genetics & Genomics, University of Georgia, Athens, GA, 30602, USA; 3 National Center for Genome Resources, Santa Fe, NM, 87505, USA

## Abstract

**Background:**

Alfalfa, a perennial, outcrossing species, is a widely planted forage legume producing highly nutritious biomass. Currently, improvement of cultivated alfalfa mainly relies on recurrent phenotypic selection. Marker assisted breeding strategies can enhance alfalfa improvement efforts, particularly if many genome-wide markers are available. Transcriptome sequencing enables efficient high-throughput discovery of single nucleotide polymorphism (SNP) markers for a complex polyploid species.

**Result:**

The transcriptomes of 27 alfalfa genotypes, including elite breeding genotypes, parents of mapping populations, and unimproved wild genotypes, were sequenced using an Illumina Genome Analyzer IIx. *De novo* assembly of quality-filtered 72-bp reads generated 25,183 contigs with a total length of 26.8 Mbp and an average length of 1,065 bp, with an average read depth of 55.9-fold for each genotype. Overall, 21,954 (87.2%) of the 25,183 contigs represented 14,878 unique protein accessions. Gene ontology (GO) analysis suggested that a broad diversity of genes was represented in the resulting sequences. The realignment of individual reads to the contigs enabled the detection of 872,384 SNPs and 31,760 InDels. High resolution melting (HRM) analysis was used to validate 91% of 192 putative SNPs identified by sequencing. Both allelic variants at about 95% of SNP sites identified among five wild, unimproved genotypes are still present in cultivated alfalfa, and all four US breeding programs also contain a high proportion of these SNPs. Thus, little evidence exists among this dataset for loss of significant DNA sequence diversity from either domestication or breeding of alfalfa. Structure analysis indicated that individuals from the subspecies *falcata*, the diploid subspecies *caerulea*, and the tetraploid subspecies *sativa* (cultivated tetraploid alfalfa) were clearly separated.

**Conclusion:**

We used transcriptome sequencing to discover large numbers of SNPs segregating in elite breeding populations of alfalfa. Little loss of SNP diversity was evident between unimproved and elite alfalfa germplasm. The EST and SNP markers generated from this study are publicly available at the Legume Information System ( http://medsa.comparative-legumes.org/) and can contribute to future alfalfa research and breeding applications.

## Background

Alfalfa, a forage legume with highly nutritious biomass, is one of the most widely planted crops in the United States, with about 21 million acres for hay production and a total value of $10 billion (USDA, online resource http://www.nass.usda.gov). Alfalfa has also been proposed as a dual-purpose biofuel crop, in which stems could be separated from leaves and used as biofuel feedstocks, with the leaves used for feeding animals [1].

Cultivated alfalfa is a tetrasomic tetraploid and allogamous; it is commercialized as synthetic populations. Simply inherited traits with high heritability have been greatly improved in alfalfa cultivars using traditional phenotypic selection, but improvement in complex quantitatively inherited traits like biomass yield or persistence has been less successful. Marker assisted selection (MAS) could accelerate alfalfa improvement [2]. Framework genetic linkage maps have been constructed for diploid and tetraploid alfalfa using a limited number of AFLP, RFLP, and SSR markers [3-7], and QTL associated with agronomic traits have been identified, albeit within relatively large genomic regions [8-12].

The availability of a large number of SNP markers and cost-effective genotyping assays facilitates the genetic dissection of complex traits via genome-wide association studies (GWAS). Many QTL related to agronomic traits in various crops have been located in genetically diverse populations using GWAS [13-16]. Using transcriptome sequencing, or RNA-seq, large numbers of SNPs have been identified recently within and between two highly divergent alfalfa genotypes [17,18] and a high resolution melting technology has been implemented to assay individual SNP markers in tetraploid alfalfa [19]. While these experiments identified numerous SNPs that can be used as markers, the source genotypes were not derived from elite breeding populations, and one of the individuals was derived from subsp. *falcata*, which is highly differentiated at the DNA marker level from cultivated subsp. *sativa*[20]. Therefore, further transcriptome sequencing of a broader array of germplasm, including diverse elite breeding genotypes, could identify SNPs that would be more useful in modern alfalfa breeding programs.

Our objective in this experiment was to sequence the transcriptomes of diverse alfalfa genotypes including 16 from four commercial USA breeding programs, six parents of mapping populations, and five representatives of wild germplasm, in order to identify and validate SNPs among the germplasm and to use the results to assess broad patterns of diversity among the genotypes. We answered several questions: (1) what proportion of SNPs identified in wild or non-elite cultivated genotypes have been retained in elite breeding pools; (2) is high genetic variation and diversity maintained in cultivated genotypes and within each commercial breeding program; and (3) what proportion of the genetic variation and diversity is shared among different breeding programs?

## Results

### De novo assembly and annotation

Between 17.2 and 31.6 million 72-bp reads were obtained for each of the 27 transcriptomes (Table 1). *De novo* assembly of quality-filtered reads generated 25,183 contigs with a total length of 26.8 Mbp (covering ~ 3.4% of the expected 800 Mbp alfalfa genome), giving an average read depth of 55.9-fold for each genotype. The length of the contigs ranged from 100 to 12,860 bp, with an average length of 1,065 bp. About 92% of the contigs were longer than 250 bp (Table 2).

**Table 1:** Description of the 27 alfalfa genotypes used in this study, their approximate fall dormancy level, and overall sequence statistics

**Entry**	**Group**^**†**^	**Source or Reference**^**‡**^	**Ploidy**	**Subspecies**	Approximate fall dormancy level*	**Total reads (millions)**	**Reads aligned (%)**	**Uniquely aligned reads (%)**
B75GH-402	1	FGI	4	subsp. *sativa*	4	26.5	92.9	86.3
B85-912	1	FGI	4	subsp. *sativa*	4	23.9	92.8	86.0
B85-920	1	FGI	4	subsp. *sativa*	4	29.5	92.9	86.6
B86-220	1	FGI	4	subsp. *sativa*	4	29.9	93.4	87.2
CW A-9	1	C/W	4	subsp. *sativa*	9	28.2	93.2	87.1
CW B-7	1	C/W	4	subsp. *sativa*	7	31.6	93.3	87.0
CW D-10	1	C/W	4	subsp. *sativa*	10	27.5	92.9	86.6
CW I-4	1	C/W	4	subsp. *sativa*	4	24.8	92.7	85.9
CV020017	1	Pioneer	4	subsp. *sativa*	4	28.1	92.8	86.4
DW000577	1	Pioneer	4	subsp. *sativa*	4	24.5	92.9	86.3
LH050543	1	Pioneer	4	subsp. *sativa*	3	24.9	92.8	86.4
NL002724	1	Pioneer	4	subsp. *sativa*	4	23.1	93.8	88.5
DL317	1	DL	4	subsp. *sativa*	8	28.1	92.9	86.2
DL833	1	DL	4	subsp. *sativa*	4	30.1	93.3	87.0
DL879W4	1	DL	4	subsp. *sativa*	4	27.4	93.4	86.9
DL263	1	DL	4	subsp. *sativa*	4	24.7	93.3	87.0
95-608	2	[21]	4	subsp. *sativa*	9	28.1	92.8	86.0
Altet-4	2	[21]	4	subsp. *sativa*	5	26.8	93.0	86.1
NECS-141	2	[21]	4	subsp. *sativa*	4	25.8	92.3	85.6
ABI408	2	[10-12]	4	subsp. *sativa*	3	27.3	93.2	86.8
Gabès	2	INRA, [22]	4	subsp. *sativa*	10	17.2	92.7	86.8
Magali-A	2	INRA, [22]	4	subsp. *sativa*	6	18.8	92.3	85.2
PI243225-A	3	NPGS	2	subsp. *caerulea*	unknown	26.2	94.0	88.4
PI577551-B	3	NPGS	2	subsp. *caerulea*	unknown	19.5	92.7	86.0
PI631816-A	3	NPGS	2	subsp. *falcata*	unknown	25.1	92.6	86.6
PI251830-K	3	NPGS	2	subsp. *falcata*	unknown	29.6	92.3	85.8
WISFAL-6	3	[10-12]	4	subsp. *falcata*	2	26.8	93.1	86.7

^†^1 = elite cultivated genotypes from commercial USA breeding programs; 2 = non-elite cultivated genotypes; 3 = non-cultivated, wild germplasm.

^**‡**^FGI =Forage Genetics International; C/W = Cal/West Seeds; Pioneer = Pioneer Hi-bred, Inc.; DL = Dairyland, Inc.; INRA = French National Institute for Agricultural Research; NPGS = National Plant Germplasm System, United States Department of Agriculture.

^*^Fall dormancy based on the scale of 1 = very fall dormant to 11 = very non-dormant [23].

**Table 2:** **Number and proportion of contigs with homology to *M. truncatula/G. max/A. thaliana/ O. sativa *gene models (E value of 1× 10**^**-10**^**) for different contig lengths**

**Length of contigs**	**Number of contigs**	**Number and proportion of contigs with hit**
100-249 bp	2,046	961 (47.0%)
250-499 bp	5,365	3,955 (73.7%)
500-749 bp	4,318	3,804 (88.1%)
750-999 bp	3,211	3,023 (94.1%)
≥1,000 bp	10,243	10,073 (98.3%)
Total	25,183	21,816 (86.6%)

To assess the representation and quality of our alfalfa assembly, alfalfa sequences were analyzed using BLASTx to search the annotated protein databases of *Medicago truncatula*, *Glycine max, Arabidopsis thaliana* and *Oryza sativa*. At an E-value threshold of 1× 10^-10^, 19,762 alfalfa contigs matched 11,663 unique protein accessions from *M. truncatula*. Alfalfa contigs matched 14,670 unique accessions from *G. max*, 11,222 from *Arabidopsis*, and 10,672 from *O. sativa*. Using BLASTx against the non-redundant GenBank protein database found 14,775 protein accessions represented by 19,039 alfalfa contigs. Overall, the above BLASTx searches enabled annotation of 21,816 (86.6%) of the 25,183 contigs (Table 2). For contigs shorter than 250 bp, only 47.0% had a hit to one of the databases, while 98.3% of the contigs longer than 1000 bp had a match (Table 2).

The 11,222 unique hits from the BLASTx search of the alfalfa contigs against the *Arabidopsis* annotated protein database were compared to the whole *Arabidopsis* genome categorization. Annotation counts were obtained for each functional category by using the gene ontology (GO) annotations tool at TAIR ( http://www.arabidopsis.org/tools/bulk/go/). The comparison (Additional file 1) showed that the contigs represented all functional categories, with a similar pattern as *Arabidopsis*.

### SNP identification and validation

Consensus contigs developed across all 27 genotypes served as the basis for alignment to detect SNP. The individual sequence reads, realigned to the consensus contigs, enabled detection of 872,384 SNPs and 31,760 InDels, based on a total uniquely aligned read number > 20 and a contingency test *p*-value < 0.01. Under conditions of 90% identity and 80% coverage, 65% of the contigs aligned to the *M. truncatula* pseudomolecules (Version 3.5.1). A total of 604,164 SNPs and InDels were identified among the alfalfa sequences from those contigs, which were widely distributed along the eight *Medicago* chromosomes (Additional file 2).

**Figure 1:**
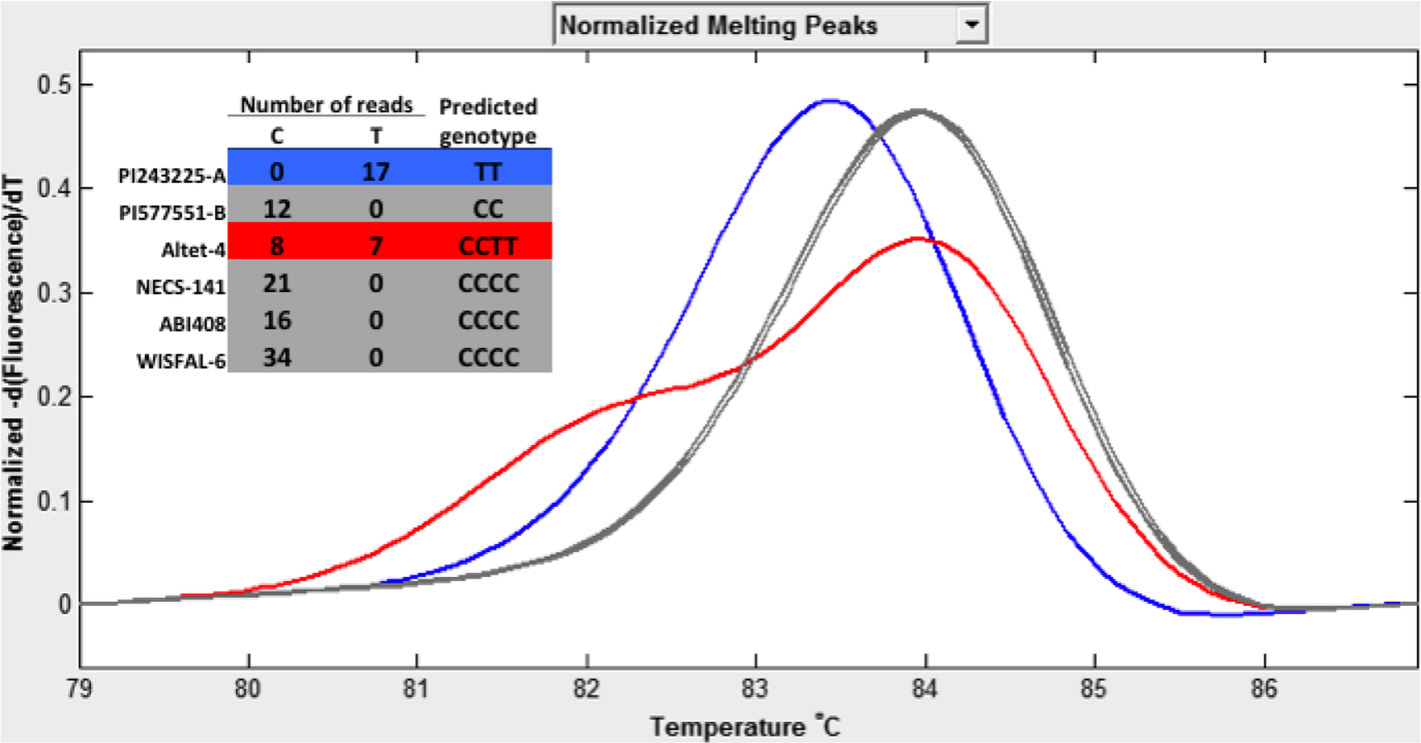
**An example of high resolution melting analysis to confirm a putative SNP.** The number of sequence reads for the two SNP allele variants is given for each of six genotypes and the predicted genotype from sequencing corresponds to the peak resulting from HRM analysis.

We selected 192 putative SNPs distributed throughout the genome to validate our results. For each of these SNPs, we developed flanking PCR primers, amplified fragments with PCR on six of the sequenced genotypes (2 diploids and 4 tetraploids), and used HRM analysis [17,19] to assess genotypes. Of the 192 SNP primers evaluated, 179 (93%) produced clear amplifications. We classified the SNP genotype for each individual as homozygous for the reference allele, homozygous for the variant allele, or heterozygous without distinguishing allelic dosages. The three genotypes were clearly distinguished based on the shape of the HRM curve (Figure 1). A comparison between the HRM profiles for the SNP markers and the sequencing results indicated that 163 of the 179 (91%) successfully amplified samples had melting profiles in agreement with the sequence results for all six genotypes.

### Heterozygosity, diversity, and population structure among 27 genotypes

In order to compare various metrics across genotypes, we retained sequences represented by at least ten sequence reads in each of the 27 genotypes. In this group, we identified 173,947 SNPs and 4,033 InDels. For each SNP, a given genotype was scored as homozygous for the reference allele (AA), homozygous for the variant allele (BB) or heterozygous (AB) which included all three potential heterozygous classes in the tetraploids (ABBB, AABB, and AAAB). The heterozygosity of the 22 cultivated tetraploid *sativa* genotypes ranged from 35.2% to 50.4%, except for two genotypes, Gabès and Magali-A, which had heterozygosities of about 30%. The non-cultivated tetraploid *falcata* WISFAL-6 had a heterozygosity of 36.2% (Additional file 3). For diploid alfalfa genotypes, heterozygosity ranged from 21.2 to 28.2% (Additional file 3). Considering the sequences from the two diploid genotypes of each subspecies as one group (i.e., four chromosomes, equivalent to a tetraploid), heterozygosity was 40.1% for *caerulea* and 42.9% for *falcata* (Additional file 3).

**Figure 2:**
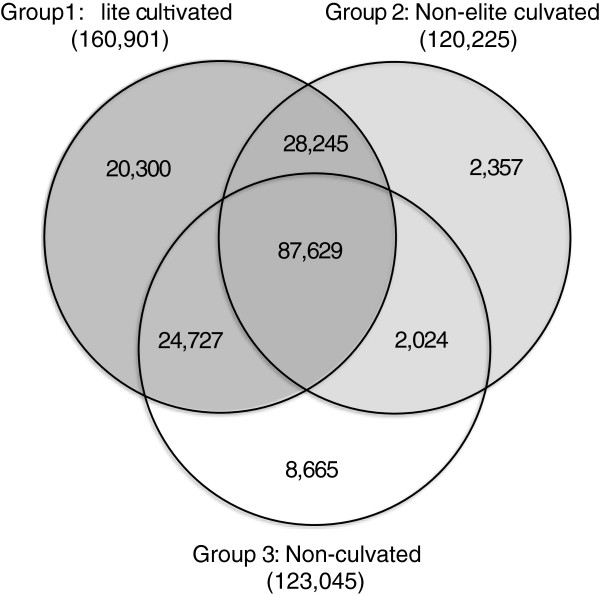
**Venn diagram showing the number of SNP segregating within three groups of alfalfa genotypes.** Numbers are based on a total number of SNP equal to 173,947 for which sequence information from all 27 genotypes was available.

To investigate the extent of SNP diversity that has been lost during domestication and breeding, we compared the number of SNP segregating in each of the three groups. Of the 173,947 SNPs, 92.5% showed polymorphism within the 16 elite cultivated tetraploid genotypes (Group 1) and 95% showed polymorphism within both cultivated pools (Groups 1 and 2) (Figure 2 and Table 1). Only 7% (8,665) of the SNPs that were present within or among the five non-cultivated genotypes (Group 3) were not polymorphic within the other two groups (Figure 2 and Table 1). No SNP locus was fixed for different alleles between any two of the three groups.

Although our genotypic sampling was limited, we investigated the SNPs which were present in the non-cultivated genotypes but fixed in the cultivated genotypes for their potential involvement in either domestication or speciation. First, to identify candidate genes possibly associated with selection during domestication and/or breeding, we identified 2,631 SNPs of the total 8,665 that were polymorphic within or between the two *caerulea* genotypes (and possibly also polymorphic in the *falcata* genotypes) but fixed in cultivated genotypes. Of these 2,631 SNPs, 94 were clustered into 15 contigs each containing at least five SNPs and could represent domestication genes (Additional file 4). One gene, a putative ATP-dependent RNA helicase, contained twelve SNPs within the gene among the four chromosomes of *M. caerulea*, yet all twelve were fixed for one allele in all cultivated alfalfa genotypes. The potential role of this gene in domestication is not obvious to us, however.

Second, of the 8,665 SNPs present only in the five non-cultivated genotypes, 6,034 were polymorphic only in the three *falcata* genotypes but fixed in all 24 *sativa* and *caerulea* genotypes. Of these, 677 SNP were clustered into 111 contigs that contained at least five SNPs. The genes represented by these contigs could possibly be related to the evolutionary differentiation between *falcata* and *sativa/caerulea* (Additional file 5).

**Figure 3:**
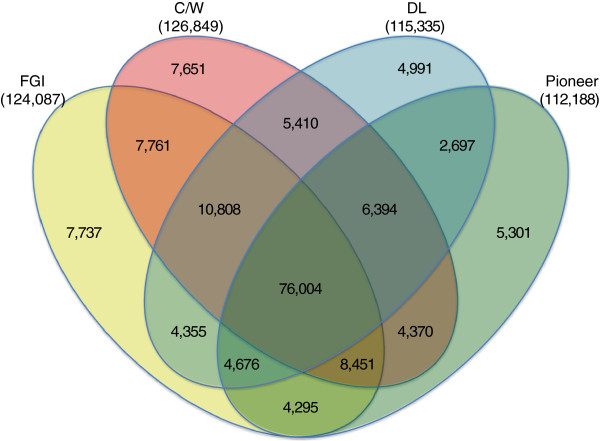
**Numbers of SNP that showed polymorphism within groups of four genotypes derived from four commercial breeding programs.** Numbers in the Venn diagram are based on 160,901 SNP polymorphic within 16 elite cultivated genotypes from four commercial breeding programs. FGI = Forage Genetics International; C/W = Cal/West Seeds; Pioneer = Pioneer Hi-bred, Inc.; DL = Dairyland, Inc.

Of the 160,901 SNPs polymorphic within the elite 16 cultivated genotypes, 124,087 (77.1%) showed polymorphism within the Forage Genetics International (FGI) subgroup of four genotypes, 112,188 (69.7%) for the Pioneer Hi-bred, Inc. (Pioneer) subgroup, 126,849 (78.8%) for the Cal/West Seeds (C/W) subgroup, and 115,335 (71.7%) for Dairyland, Inc. (DL) subgroup (Figure 3). Although some (3-5%) SNPs were segregating within only one subgroup, nearly half (47%, 76,004 SNPs) were segregating in all four subgroups (Figure 3).

**Figure 4:**
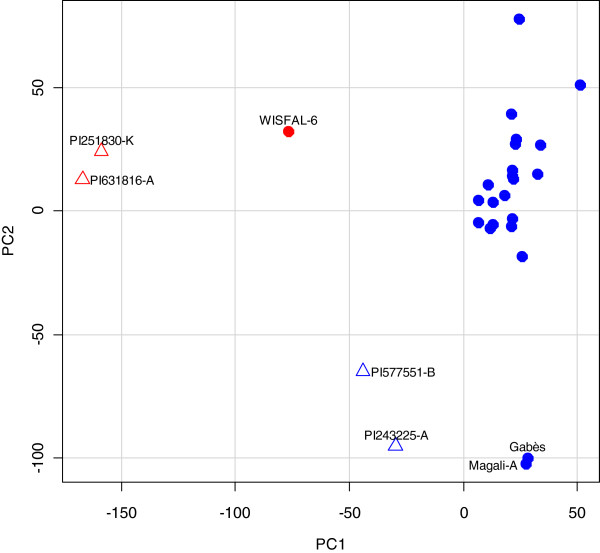
**Plot of the first two principal components from a principal components analysis of SNP variation among 27 alfalfa genotypes.** Blue solid circles represent tetraploid *sativa*; red solid circle represents tetraploid *falcata*; blue triangles represent diploid *caerulea*; red triangles represent diploid *falcata.*

**Figure 5:**
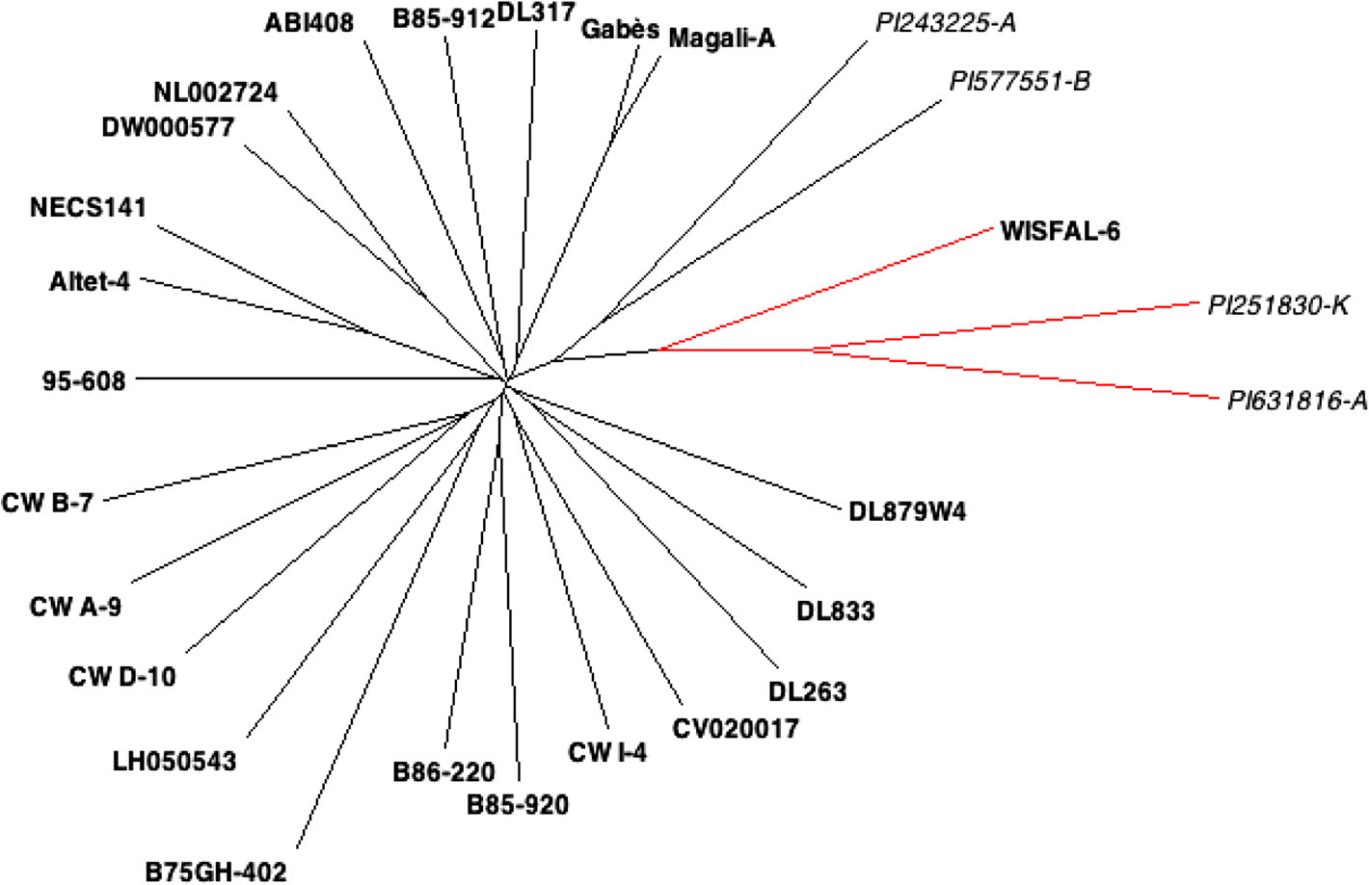
**A neighbor-joining phylogenetic tree of 27 alfalfa genotypes based on SNP variation.** Red line with a bold label represents tetraploid *falcata*; red lines with italic labels represent diploid *falcata*; black lines with bold labels represent tetraploid *sativa*; black lines with italic labels represent diploid *caerulea.*

We assessed population structure based on the 173,947 SNPs using principal components analysis (PCA). The first PC, explaining 9.97% of the total variance, clearly separated the tetraploid *sativa*, diploid *caerulea*, diploid *falcata*, and tetraploid *falcata* genotypes (Figure 4). The second PC explained 6.70% of the total variance and distinguished the French genotypes Gabès and Magali-A from the cultivated tetraploid *sativa* genotypes from the US (Figure 4). A similar result was obtained from phylogenetic analysis (Figure 5), in which genotypes from subspecies *falcata* clearly separated from subspecies *caerulea* and *sativa*. The phylogenetic analysis also showed that nondormant genotypes from Cal/West Seeds are clustered, though not clearly separated from other germplasm.

## Discussion

### Assembly and annotation

In this study, 25,183 contigs were generated from *de novo* assembly of short, 72-bp transcriptome sequences derived from 27 diverse alfalfa genotypes. A high percentage (87%) of the resulting contigs matched protein accessions from other species based on a BLASTx search. The high number of matches is likely due to the fact that most (92%) of our contigs were longer than 250 bp.

In order to obtain high read depth and high quality SNPs for the genes related to cell wall and other biofuel-related traits, we only sampled stems for RNA isolation. Therefore, genes not expressed in stems could be missed. In addition, genes that are expressed at low levels, transiently, or only under specific environmental stimuli, such as under various stress conditions, are likely to be absent in our dataset. However, even though sampling was limited to alfalfa stems grown under a single environmental condition at a single time point, the GO analysis indicated that a broad diversity of genes was represented by the contigs, enabling investigation of genetic variation for candidate genes involved in expression of many different traits. We are in the process of identifying SNPs in candidate genes for cell well composition, cold tolerance, flowering time, and other traits, designing marker assays, and mapping them in segregating populations.

Previous sequencing of transcriptomes of two tetraploid alfalfa genotypes using a 454 GS-FLX instrument generated 24,144 tentative consensus sequences (TCs), matching 15,268 unique protein accessions of *M. truncatula* at an E value cutoff of 1× 10^-5^[17]. Reevaluating our results at the same E-value identified 20,966 contigs matched to 12,019 unique protein accessions. Combining both studies identified 17,011 unique protein accessions, with 10,276 identified in both experiments. Collectively, these experiments and others undoubtedly in progress will lead to a highly robust dataset for alfalfa that will enable SNP identification throughout the genome in virtually any gene of interest, making the tagging of specific genes (and genic regions) with molecular markers a reality.

### SNP discovery, validation, and potential applications

After potential false-positive SNPs were removed by filtering out samples that had inadequate read depth, that failed a contingency test, and that had low quality scores, we identified 872,384 SNPs and 31,760 InDels among the 27 genotypes. We obtained a validation rate of 91% for the predicted SNPs using HRM analysis, a higher validation rate of SNPs identified from next generation transcriptome sequencing than has been observed in other experiments, which ranged from 66% to 85% [24-27]. We only attempted to validate SNPs that had flanking regions of at least 18 bp free of other polymorphisms on both sides, so it’s possible that the validation rate of 91% is higher than other studies because of this requirement. Most of the HRM phenotypes that did not match the inferred sequence-based genotype were assigned as putative homozygotes from sequencing but observed as heterozygotes with HRM (data not shown). This result can be explained by allele specific expression and/or low sequence read depth in some alfalfa genotypes.

In this study, we extended previous alfalfa SNP discovery experiments by sequencing a large number of genotypes including elite genotypes from four major US alfalfa breeding companies. Our goal was to identify SNP markers that would be useful in contemporary breeding programs. The high proportion of SNPs identified across all 27 genotypes that segregated within or among elite cultivated genotypes from the four commercial breeding programs suggests that little genetic variation has been lost during domestication and the past 50–100 years of breeding. The high proportion of SNPs showing polymorphism even within the four genotypes from each breeding program suggests large effective population sizes and relatively weak bottlenecks imposed by breeding. Commercial breeding populations have been in existence for about 100 years, but the most substantial selection intensity has probably occurred over the past 40 years, and the actual number of cycles of selection during that time is probably on the order of 8–12 generations, due to multiyear evaluations necessary for perennial alfalfa. This probably contributes to the preservation of high genetic variation and diversity. Only 3-5% of the SNPs segregating in elite germplasm were polymorphic within only a single breeding program. Three of four genotypes from the Cal/West subgroup were non-dormant, but they did not show a different pattern compared to other groups (Figure 3). This suggests that germplasm brought to the United States at various times has been considerably mixed between dormant and non-dormant germplasm groups and perhaps shared among different breeding programs.

Previous population structure analyses have indicated clear differentiation of diploid subspecies *falcata* from diploid subspecies *caerulea*[28] and of tetraploid subspecies *falcata* from tetraploid subspecies *sativa* germplasm introduced into North America [20,29]. Based on the many SNPs identified in this experiment, we also differentiated individuals from subspecies *falcata*, subspecies *caerulea*, and subspecies *sativa* (cultivated tetraploid alfalfa). Heterosis for yield and other agronomic traits have been found in hybrids derived from the two subspecies [30-33]. Previously, favorable alleles have been identified in wild diploid and tetraploid alfalfa germplasms [10-12,34]. These results suggest that potential complementary favorable alleles or valuable alleles for specific traits may exist in subspecies *falcata* and diploid subspecies *caerulea*, and if integrated into elite cultivated alfalfa by marker-assisted breeding could improve current cultivars.

An anomaly in the current results is the close relationship between Gabès and Magali-A, genotypes which have been previously shown to be quite divergent (B. Julier, INRA, Lusignon, France, pers. comm.). We pooled stem samples from three selfed progeny individuals for each of the genotypes, and perhaps this explains the somewhat lower heterozygosity values of the genotypes. However, that does not explain the close relationship shown in the PC plot. As a mixture of individuals would be expected to result in higher heterozygosity, that explanation does not appear to be likely. Thus, we are not able to explain this result, and consequently, the close relationship between these genotypes should be viewed skeptically.

Molecular markers are useful for linkage map construction and QTL mapping. Although complex tetrasomic inheritance, heterozygosity, and lack of inbred lines due to inbreeding depression constrain genetic map construction and QTL analysis in tetraploid alfalfa, the availability of large numbers of markers and a time/cost effective genotyping assay can overcome these limitations. In the present study, seven of 27 sequenced genotypes are parents of diploid and tetraploid mapping populations. A large number of SNPs that are polymorphic between parents can be used to saturate previously constructed framework linkage maps from those bi-parental segregating populations and facilitate QTL and candidate gene mapping relevant to agronomic traits.

We evaluated the SNP distribution and genome coverage based on the whole genome sequence of *M. truncatula*[35], a close relative of alfalfa. A high level of synteny between alfalfa and *M. truncatula* has been found in previous comparative studies [4,36]. The SNPs are widely distributed along all eight chromosomes (Additional file 2). In addition, selected SNPs from this study have been mapped to genetic linkage maps and their mapped locations matched physical location orders (data not shown). These genome-wide SNPs can be applied to genome wide association studies (GWAS) to capture unknown QTL and to accelerate marker assisted breeding.

## Conclusion

Transcriptome sequencing identified a large number of SNPs relevant to commercial alfalfa breeding programs. The amount of SNPs variation in elite commercial breeding programs has not diminished substantially from wild alfalfa, suggesting few if any genetic bottlenecks in the history of alfalfa domestication and breeding. The sequence results can be applied to comparative genomics analyses between alfalfa and other species and will be valuable for annotating the alfalfa genome sequencing project that is currently being underway. The gene-targeted SNPs from the present study can be used for genetic diversity and QTL identification experiments, particularly by focusing marker efforts on candidate genes for important phenotypes. The genome-wide distribution of SNP will facilitate map saturation, making QTL mapping, GWAS, and genomic selection potentially feasible in alfalfa. The sequence data and SNP markers will be valuable genomic resources for future alfalfa research and breeding applications.

## Methods

### Plant materials

A total of 27 alfalfa genotypes were used in this study and divided into three groups (Table 1). Group 1 consisted of 16 elite tetraploid *sativa* genotypes from four commercial breeding programs: CW A-9, CW B-7, CW D-10, and CW I-4 from Cal/West Seeds (C/W); B75GH-402, BG85-912, BG85-920, and BG86-220 from Forage Genetics International (FGI); CV020017, DW000577, LH050543, and NL002724 from Pioneer Hi-bred, Inc. (Pioneer); DL263, DL317, DL833, and DL879W4 from Dairyland, Inc. (DL), with a range of fall dormancy levels (Table 1). Group 2 includes six cultivated tetraploid *sativa* genotypes used as parents for mapping populations but not elite, commercial genotypes (ABI408 is one parent of a population used for mapping yield and other traits [10-12]; Altet-4, 95–608, and NECS-141 are parents of mapping populations used for aluminum tolerance QTL mapping [21]; and Gabès and Magali-A are parents of a mapping population developed in INRA, France [22]). The Gabès and Magali-A parental genotypes were represented in this experiment by a bulk of three selfed progeny genotypes. Group 3 includes five non-cultivated genotypes: WISFAL-6 is a tetraploid genotype from *M. sativa* subsp. *falcata* and four wild diploid alfalfa genotypes, PI243225-A (*M. sativa* subsp. *caerulea* from Iran), PI577551-B (*M. sativa* subsp. *caerulea* from Canada), PI631816-A (*M. sativa* subsp. *falcata* from Russia), and PI251830-K (*M. sativa* subsp. *falcata* from Austria), obtained from the USDA-National Plant Germplasm System ( http://www.ars-grin.gov/). WISFAL-6 is the other parent of a mapping population with ABI408 [10-12]; PI243225-A and PI577551-B are the parents of a diploid mapping population [4]. All plants were grown in the University of Georgia greenhouse in Athens, GA.

### RNA isolation

To preferentially capture genes related to cell wall composition – particularly, those involved in lignin biosynthesis – and other biofuel related traits, we sampled old and young stems for RNA isolation and sequencing. For each genotype, flowering stems were identified and two samples were taken, one representing older (and hence more lignified) tissue (the lower 3 internodes) and the other representing younger (and hence less lignified) tissue (the upper 3 internodes). Stems were sampled from 40 days-old regrowth after trimming from single plants except for Gabès and Magali-A, where young and old stem tissues were sampled from three selfed progeny plants and pooled across progeny for each tissue age. Tissues were immediately frozen in liquid nitrogen. Total RNA was isolated from frozen stem tissue with TRIzol reagent using standard procedures [37] and quantified by NanoDrop Spectrophotometer ND-100 (NanoDrop Technologies, Willington, DE). An equal amount of RNA from old and young stem samples from each genotype was pooled for cDNA library construction.

### cDNA library construction

Polyadenylated RNA was isolated from total RNA using oligo-dT25 magnetic beads (Dynabeads; Invitrogen, Carlsbad, CA), denatured, and used as a template for random-primed cDNA synthesis and end-repair with T4 DNA polymerase, Klenow polymerase and dNTPs. Polished fragments were phosphorylated by T4 polynucleotide kinase, followed by the addition of a single “A” base to the 3′-end of the blunt-ended phosphorylated DNA fragments. Illumina sequencing adapters were then added to the DNA fragments by ligation and size selected by electrophoresis for a desired size range of ~500 bp. Purified DNA libraries were amplified by PCR for 15 cycles. Libraries were qualitatively and quantitatively assessed by Nanodrop ND-1000 (Thermo Scientific, Waltham, MA) UV/Vis spectroscopy and DNA BioAnalyzer 2100 microfluidics (Agilent, Santa Clara, CA). Two picomoles of the size-selected cDNA library were loaded on an Illumina single-end flow cell using the Illumina Cluster Station (Illumina, Inc., San Diego, CA) at the National Center for Genome Resources in Santa Fe, NM.

### Sequencing

72 bp single-end reads were produced on Illumina Genome Analyzer IIx machines using sequencing-by-synthesis technology. Base calling, per-base quality scoring and initial quality filtering of read data were performed using Illumina’s RTA Image analysis software and CASAVA-1.7. Contaminant filtering against Illumina adapter sequences and other common artifacts was performed using BLAST and custom post-processing scripts by the sequencing facility. Transcriptome sequences were deposited into the short read database of the National Center for Biotechnology Information (NCBI) [accession number SRP009188].

### Sequence assembly and annotation

Reads from each alfalfa genotype were quality-trimmed and assembled separately into “synthetic ESTs” using Abyss [38] performing k-mer sweeps from 36–72 bp (using increments of 3 bp between successive k-mer runs). The resulting synthetic ESTs from these sweeps had redundancies removed via CD-HIT [39] for the purposes of computation, and then were assembled together using miraEST [40] using its strain mode, allowing it to use the strain information in each sample set to assemble through allelic differences. Short read assembly algorithm’s sensitivity to variation prevented a simple pooled approach. The resulting transcripts were assessed for level of support by read-remapping of the input 72 bp reads against the transcripts, using GSNAP [41] and subsequent alignment filtering and initial SNP calling by the Alpheus pipeline [42]. Annotation of the transcripts was performed using BLASTn and BLASTx with an 10^-10^ E-value cut-off against individual databases comprising gene models from model plant species *A. thaliana*, *G. max*, *M. truncatula*, *O. sativa*; BLASTx was performed against the NCBI nr protein database; and InterProScan [43] analysis against 6-frame translations of the transcripts. Genomic mapping of transcripts to the *M. truncatula* 3.5 genome were performed using GMAP [44], and post-filtered for identity and coverage requirements.

### SNP identification

The filters we used to call a variant a true SNP are as follows: in at least one of the 27 samples, the allele of the variant was called with at least 2 reads, at bases whose average quality is at least 20 and with 20% frequency within the reads covering the position in that sample. To remove the potential false-positive SNP identifications due to sequencing errors, we added two more filters: a total unique aligned read number greater than 20 across all samples and *p* value of genotypic contingency test less than 0.01. Genotypic contingency test was conducted for testing independence of reference and variant alleles (rows) across 27 genotypes (columns) in a contingency table of read counts, as described by Myles et al. [45].

### SNP validation

A subset of SNPs identified from sequencing that had flanking regions of at least18 bp on each side free of other polymorphism were validated using HRM assays following the protocol described by Han et al. [17,19]. Primer pairs targeting each single SNP were designed using computer program Batch Primer 3 ( http://probes.pw.usda.gov/batchprimer3/) with the following criteria: predicted annealing temperature (Tm) of 59°C to 61°C (optimal 60°C); primer length ranging from 18 to 24 bp (optimal 20°C); PCR amplicon length of 40 to 130 bp (optimal 50 bp); PCR amplicon Tm between 70°C and 87°C (optimal 78°C). In total 192 SNPs were evaluated for SNP validation using the HRM assay (Additional file 6). Six genotypes (Altet-4, NECS-141, ABI408, WISFAL-6, PI243225-A, and PI577551-B) were selected for SNP validation and their DNA was isolated from fresh leaves following the CTAB method [46]. PCR reactions were performed in 384-well format on a 9700 Thermal Cycler (Applied Biosystems, Foster City, CA, USA) in a total volume of 5 μl per reaction under 10 μl mineral oil, including 5 ng of genomic DNA, 0.25 μM of forward and reverse primer, 1X LightScanner High Sensitivity Master Mix (Idaho Technologies, Salt Lake, UT, USA) that contains LCGreen. PCR program started with denaturation at 95°C for 2 min, followed by 40 cycles of 94°C for 30 s and 65°C for 30 s, and finally held at 4°C. PCR products were scanned using a LightScanner 384-well system (Idaho Technologies, Salt Lake, UT) with a temperature gradient of 0.1°C per second in the range between 59 to 95°C. Melting data was visualized and analyzed with the LightScanner Software using the small amplicon module from CALL-IT 2.0 (Idaho Technologies, Salt Lake, UT).

### Diversity and population structure

Using SNPs identified from sequences that had at least ten reads for each of the 27 genotypes, principal components analysis (PCA) was performed using R package *labdsv*. Based on the same SNP dataset, simple matching distances between each pair genotypes were calculated using R package *ade4*. Then the matrix of estimated pairwise distances was used to construct unweighted neighbor-joining trees using the software PHYLIP version 3.67 [47]. The resulting dendrogram was plotted using the program Dendroscope [48].

## Competing interests

The authors declare that they have no competing interests.

## Authors’ contributions

XL coordinated the study. XL, ADF, and ECB wrote the manuscript. XL, AA, ADF, JAC, AKB, RSK, YW, YH, and JG analyzed data. GDM oversaw bioinformatics aspects of the experiment and MJM coordinated comparisons of the study to previous SNP work. ECB designed and supervised the entire study. All authors read, contributed to, and approved the final manuscript.

## Supplementary Material

Additional file 1 **Gene ontology assignments for *M. sativa, *compared to *A. thaliana. ***Gene ontology assignment was found for the 11,222 *Arabidopsis* best-hit gene models (1× 10^-10^). Numbers of best hits in each GO category were plotted and compared to that of all of the gene models in the *Arabidopsis* genome annotation. Click here for file

Additional file 2 **SNP distribution along the *M. truncatula *chromosomes.** The X-axis represents the genome location (Mbp) for each chromosome. The number of SNP per 1000 bp was calculated for each 0.5 million base pairs interval and plotted on the Y-axis. Click here for file

Additional file 3 The numbers of SNP homozygous and heterozygous for each genotype based on 173,947 SNPs for which sequence information was available for all genotypes. Click here for file

Additional file 4 Potential candidate genes involved with domestication and/or selection. Click here for file

Additional file 5 **Potential candidate genes differentiating subsp. *falcata *from subsp. *sativa.*** Click here for file

Additional file 6 SNP primer sequences used for validation using high resolution melting (HRM). Click here for file
