# Supplementary figures and images for: Prevalence of single nucleotide polymorphism among 27 diverse alfalfa genotypes as assessed by transcriptome sequencing
Source: BMC Genomics. 2012 Oct 29;13:568. doi: 10.1186/1471-2164-13-568 (PMC3533575; doi:10.1186/1471-2164-13-568)

**Additional file 1** Gene ontology assignments for *M. sativa* and *A. thaliana*


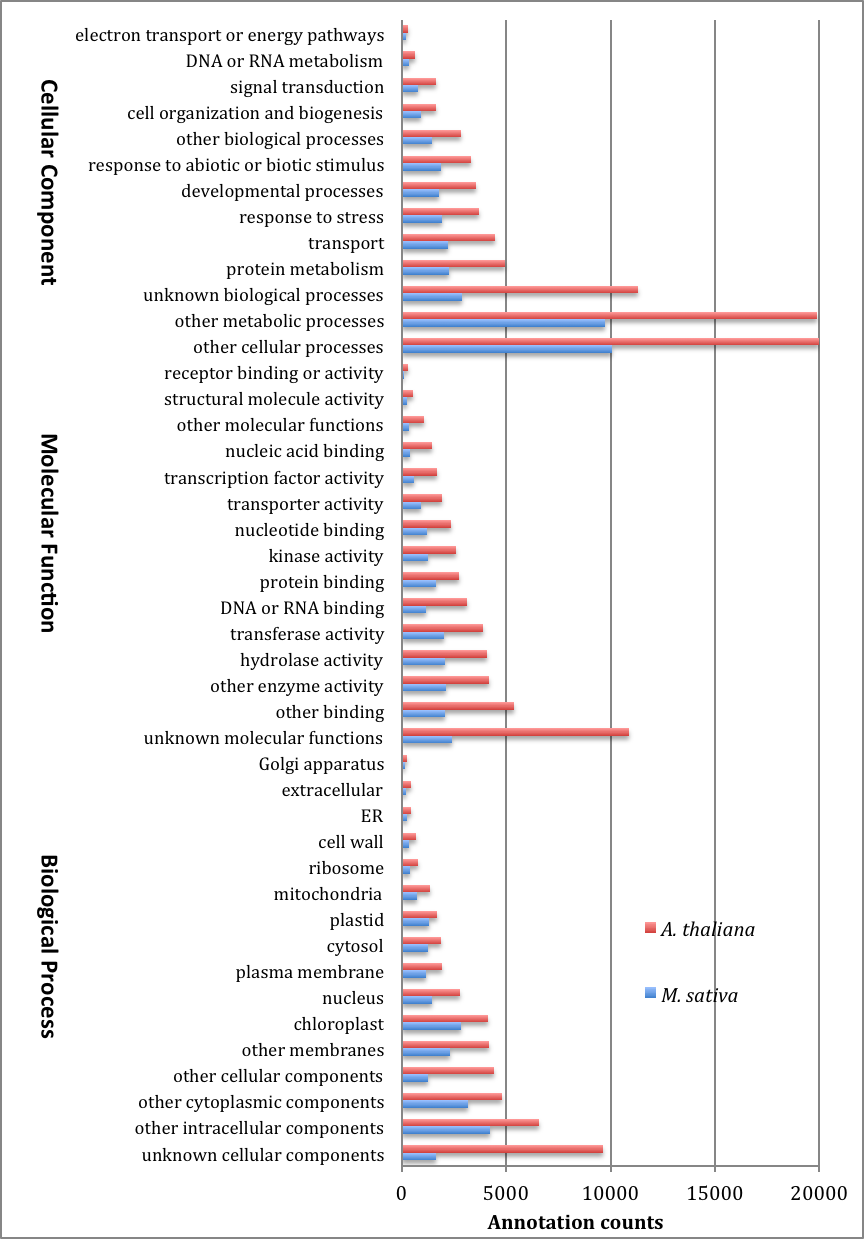

Supplement: Additional file 1 — Gene ontology assignments for M. sativa, compared to A. thaliana. Gene ontology assignment was found for the 11,222 Arabidopsis best-hit gene models (1× 10^-10^). Numbers of best hits in each GO category were plotted and compared to that of all of the gene models in the Arabidopsis genome annotation. [file 1471-2164-13-568-S1.doc]
