# Supplementary material for: Prevalence of single nucleotide polymorphism among 27 diverse alfalfa genotypes as assessed by transcriptome sequencing
Source: BMC Genomics. 2012 Oct 29;13:568. doi: 10.1186/1471-2164-13-568 (PMC3533575; doi:10.1186/1471-2164-13-568)

**Additional file 2** SNP distribution along the *M. truncatula* chromosomes*.* The X-axis represents the genome location (Mbp) for each chromosome. The number of SNP per 1000 bp was calculated for each 0.5 million base pairs interval and plotted on the Y-axis.


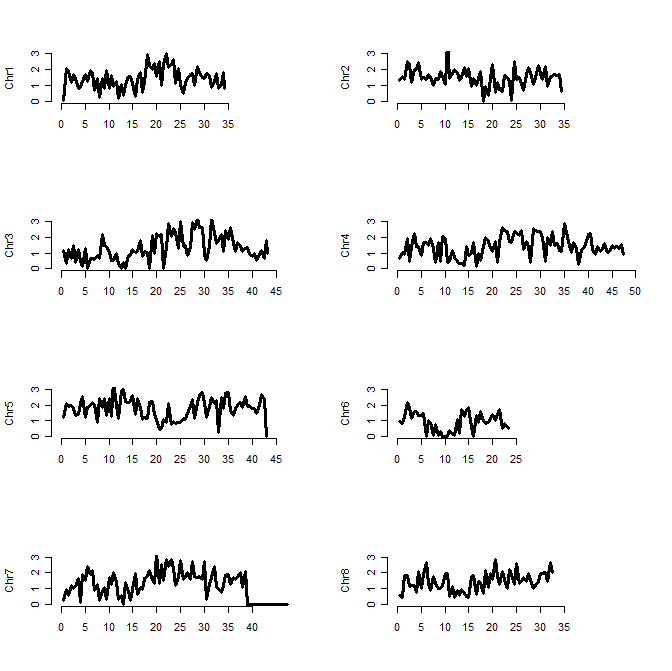

Supplement: Additional file 2 — SNP distribution along the M. truncatula chromosomes. The X-axis represents the genome location (Mbp) for each chromosome. The number of SNP per 1000 bp was calculated for each 0.5 million base pairs interval and plotted on the Y-axis. [file 1471-2164-13-568-S2.doc]
