# Supplementary material for: Prevalence of single nucleotide polymorphism among 27 diverse alfalfa genotypes as assessed by transcriptome sequencing
Source: BMC Genomics. 2012 Oct 29;13:568. doi: 10.1186/1471-2164-13-568 (PMC3533575; doi:10.1186/1471-2164-13-568)
Supplement: Additional file 3 — The numbers of SNP homozygous and heterozygous for each genotype based on 173,947 SNPs for which sequence information was available for all genotypes. [file 1471-2164-13-568-S3.doc]

**Additional file 3** The numbers of SNP that are homozygous and heterozygous for each genotype, based on 173,947 SNPs for which sequence information is available for all 27 genotypes.

| **Genotype** | **Homozygous** | **Heterozygous** | **Heterozygosity %** |
| --- | --- | --- | --- |
| B75GH-402 | 88,035 | 75,902 | 46.3 |
| B85-912 | 112,640 | 61,307 | 35.2 |
| B85-920 | 99,198 | 74,749 | 43.0 |
| B86-220 | 108,889 | 65,058 | 37.4 |
| CW A-9 | 100,406 | 73,541 | 42.3 |
| CW B-7 | 86,221 | 87,726 | 50.4 |
| CW D-10 | 99,226 | 74,721 | 43.0 |
| CW I-4 | 104,995 | 68,952 | 39.6 |
| CV020017 | 106,510 | 67,437 | 38.8 |
| DW000577 | 108,970 | 64,977 | 37.4 |
| LH050543 | 110,242 | 63,705 | 36.6 |
| NL002724 | 111,708 | 62,239 | 35.8 |
| DL317 | 111,629 | 62,318 | 35.8 |
| DL833 | 107,749 | 66,198 | 38.1 |
| DL879W4 | 107,262 | 66,685 | 38.3 |
| DL263 | 100,136 | 73,811 | 42.4 |
| 95-608 | 112,094 | 61,853 | 35.6 |
| Altet-4 | 105,009 | 68,938 | 39.6 |
| NECS-141 | 101,272 | 72,675 | 41.8 |
| ABI408 | 108,532 | 65,415 | 37.6 |
| Gabès | 120,329 | 53,618 | 30.8 |
| Magali-A | 122,687 | 51,260 | 29.5 |
| PI243225-A | 137,015 | 36,932 | 21.2 |
| PI577551-B | 126,137 | 47,810 | 27.5 |
| PI631816-A | 129,902 | 44,045 | 25.3 |
| PI251830-K | 124,883 | 49,064 | 28.2 |
| WISFAL-6 | 111,031 | 62,916 | 36.2 |
| PI243225-A+ PI577551-B | 104,190 | 69,757 | 40.1 |
| PI631816-A+ PI251830-K | 99,318 | 74,629 | 42.9 |
