# Supplementary material for: Prevalence of single nucleotide polymorphism among 27 diverse alfalfa genotypes as assessed by transcriptome sequencing
Source: BMC Genomics. 2012 Oct 29;13:568. doi: 10.1186/1471-2164-13-568 (PMC3533575; doi:10.1186/1471-2164-13-568)
Supplement: Additional file 6 — SNP primer sequences used for validation using high resolution melting (HRM). [file 1471-2164-13-568-S6.doc]

**Additional file 6 SNP primer sequences used for validation**

| Primer name | SNP ID | Forward 5'-3' | Forward Tm | Reverse 5'-3' | Reverse Tm |
| --- | --- | --- | --- | --- | --- |
|  |  |  | (^o^C) |  | (^o^C) |
| MSB193 | D_11004 | TCGGAGTCACGATCCTTTTC | 60.2 | CGGTGAATTTTCTAGCAAGTGG | 61.0 |
| MSB194 | D_297835 | AGGATCAACTGCTTTCTTTCTACC | 59.4 | TCGAAGCTTCTCTAAGGGGTATC | 60.2 |
| MSB195 | D_331786 | TGGCGAATAAAGAAAGGAAGAG | 59.9 | ACCATCTTCATCGTCTACTTGTTG | 59.6 |
| MSB196 | D_347825 | AGGAAGGGGATGCTTTAATTG | 59.5 | TTGAAGAGCACGCTCCAAG | 60.3 |
| MSB197 | D_364391 | GGAATTAAGGTAAAATGGACATGG | 59.9 | CCAGATGAACCAGGACTTGAC | 59.6 |
| MSB198 | S_10101316 | ACGTTGGGAACTTTGATTGG | 59.8 | TGTGGACATCATTGAACTACGG | 60.8 |
| MSB199 | S_10150573 | GCATACACGAGCACATCAGG | 60.3 | TCTCATCATATGCCGCTTTG | 59.8 |
| MSB200 | S_10150722 | GCCCAAAATAATGCATCAGC | 60.4 | CTGTGTTGCCAAACTAAGATCG | 59.8 |
| MSB201 | S_10460696 | GATTACCATGTGGAGCAATCC | 59.3 | AGTGGTGGAGGGACTTTCAG | 59.2 |
| MSB202 | S_10465226 | TGCTGCTGTTGTTGAGGAAC | 60.0 | CAATGCTTCCCAGAGACTGC | 60.9 |
| MSB203 | S_10657337 | TTCCACTCTTCTGGACTAACCTTC | 60.2 | AAACAACGCGTTCAGTAGACG | 60.4 |
| MSB204 | S_10817006 | CAGTCTGGTCTTTCGCAATG | 59.4 | GTCGAGCAAGAGCAAAGAGC | 60.4 |
| MSB205 | S_10817989 | GGAGTATCAACTCCAGAGCTGTC | 59.4 | CTCTTCCAAAGCAATAAACAACAG | 59.4 |
| MSB206 | S_11071629 | ATTTGAAGCCCAACAACTGC | 60.1 | GCAAGTCACTTTCCATCTCCTC | 60.3 |
| MSB207 | S_11074880 | AGTGACTCCGCCTTCTTCAG | 59.6 | TAGATGCGCCATCTGCTTC | 60.1 |
| MSB208 | S_11412322 | TCTGCATACCATCTCCTGATTC | 59.2 | TGCTTTGGCTGCTGATAGG | 60.1 |
| MSB209 | S_11699439 | GACGACGAAGACGGTTGAG | 59.4 | AGAAGAAGAAACAACCCAAGCTAC | 59.4 |
| MSB210 | S_11760034 | AAGAAGGGTCAATTGGATGAAC | 59.3 | CAAACATGACAATGTGGAAGC | 59.0 |
| MSB211 | S_11760989 | TGTTGCTTGATATCGTGATGC | 59.7 | TGGCATCATAAACGACGATG | 60.5 |
| MSB212 | S_12288657 | CCCATTAACACAGCAAGTGG | 59.1 | TGGGTTATTTTGTGGGAAGC | 59.8 |
| MSB213 | S_1231858 | GAAGTCCATTAGCCCCTTGG | 60.8 | AGGCTGCCTTGTCTGCAC | 60.2 |
| MSB214 | S_1231927 | AGCAGGTGGTGCAGACAAG | 60.0 | GCTTTCAAAGGAAGTTGCTAAAAG | 60.0 |
| MSB215 | S_1246791 | TCACATGGTGGAGTTCTTGC | 59.7 | AGCTGTCGCAACGATGTTC | 60.0 |
| MSB216 | S_12488859 | ACCCTTTTTAAGTCGCATGG | 59.1 | CTGTGTGGAAGGTGCTGAAG | 59.5 |
| MSB217 | S_12489739 | TGCTGATGGTCATTTATCACG | 59.6 | TCTTTCCTTCTCTTTACCTCCATC | 59.3 |
| MSB218 | S_12490650 | CATTGAACGCCTATATGGTGAC | 59.4 | TTACAGGCAGAGCATGAGTTG | 59.1 |
| MSB219 | S_12711608 | CGGCCATGAGTCATAATGC | 60.1 | GGCTATTGGTGGTTGAAAGTG | 59.5 |
| MSB220 | S_12711635 | AACCACCAATAGCCTCTCTCC | 59.6 | AACGATCGCAGGTCTTTGAC | 60.3 |
| MSB221 | S_13174206 | GAGCTATTGATTTGGAAGAGTCG | 59.4 | TCTGTGCTTGCGAGGTAATC | 59.0 |
| MSB222 | S_13174791 | AGGGTTCGGGTAATTGTAATACTG | 59.6 | TGTTTGCTCTTCAGTGTGGTG | 59.9 |
| MSB223 | S_13263375 | CAAGCCTGTGCTAGGTCCAC | 60.9 | GCGTGGGGCCTATTTCTC | 60.6 |
| MSB224 | S_13264009 | ACTGCTGCACCTCTTGCTC | 59.3 | TTTCTCGACACTCCTGGTCAC | 60.3 |
| MSB225 | S_13264214 | TGGTTTCTACTGCCTTTTCTCAC | 59.8 | GTGATGCAGCGTATGGTTTG | 60.1 |
| MSB226 | S_13380037 | CTGAAATGTCAGCTGTCAGAGG | 60.1 | CTGCACCATTTTGCGAGTC | 60.4 |
| MSB227 | S_13381087 | CAAAATGATGAAGGGGATGC | 60.3 | AGCCACACCTTCAGGATAGAAC | 59.6 |
| MSB228 | S_13572025 | AAGGGGTAGCAAGCTATCAGG | 59.8 | CAGACTCAAAGGCCACAATG | 59.3 |
| MSB229 | S_13572756 | CCGGATCAAAAACATTAGCC | 59.4 | ACCCTGGTGTTCAGATTGATG | 59.8 |
| MSB230 | S_13638032 | CAAGTGGTGCGAGTAGATCG | 59.5 | CCCTTCTTTGATAAAGATGCTACC | 59.6 |
| MSB231 | S_14025175 | ACCATGGATGACAGCTTGTATG | 59.9 | CGGAGTCTTGGTCAGATTCG | 60.8 |
| MSB232 | S_14028533 | CAGGAGATCCTCTGGACAAAAG | 60.2 | AGATGCTGGATTGGTGCAG | 59.8 |
| MSB233 | S_14196366 | TCCATGTTAGATCCATCAAAACC | 60.1 | GGAGAGATGATGCCAGAAGC | 59.9 |
| MSB234 | S_14196899 | CCTTCCCCACAGCATTCTC | 60.6 | GGCCAAAATGAGGAGGTAGG | 60.8 |
| MSB235 | S_14490291 | CAACATTCTCCCACAATACCC | 59.2 | AGGTCGAGGATGAAGAGTGC | 59.4 |
| MSB236 | S_14491862 | CCTGGCTATGGCTATTCCTG | 59.7 | AGAGCAACGCATGGCTTATC | 60.4 |
| MSB237 | S_14493417 | TTCCTGGCTCCTATCCTTCTG | 60.7 | TGGCAAATATCTGTGTTTGTCG | 60.9 |
| MSB238 | S_14882867 | TCGAACCTCTCCCGTTTG | 59.8 | GAGGTGTTGTCCATGCTCTG | 59.3 |
| MSB239 | S_15095675 | TGCTATGCTCTTTAATCCGTTTG | 60.6 | CGATGTGGAGAAAGAGTGAGG | 59.9 |
| MSB240 | S_15096030 | CGATCCGATTAAGGTTACACTTC | 59.1 | AGTCGTCTGCCATTTCTTCC | 59.3 |
| MSB241 | S_15194868 | TGTGACTTGCATTGGGAGAC | 59.7 | AGCTTGCAGAAGAATCAAACG | 59.7 |
| MSB242 | S_15446587 | CAGTTGGATTTTGGAGAAGAGC | 60.2 | CTCACAGCTGGTCAGAACAAAG | 60.1 |
| MSB243 | S_15518038 | GAAGAAAGCAATCAGAAACTTGC | 59.6 | AATGAGTGTATTCCCGACAGC | 59.1 |
| MSB244 | S_15518743 | GGATGTCATGGAAGCAAGG | 59.0 | GCTCATCAAGAGATGATTTCAAAG | 59.4 |
| MSB245 | S_15560749 | CCTGATATCAACATCGCACTTC | 59.6 | TGATAGGAATGGAAGCAGCAG | 60.4 |
| MSB246 | S_15561037 | GGACATCTGAAACGCTCGTC | 60.8 | AGCTACGAATTGCCTGATGG | 60.2 |
| MSB247 | S_15584148 | TCAGAATGGGAAGTTGACTGG | 60.1 | GTTCACCAGAAGGAGCAAGC | 60.0 |
| MSB248 | S_15584355 | CTGTGGCAGCAGATGGAG | 59.0 | GTAGGTAGGGGTGGTTTTGATG | 59.6 |
| MSB249 | S_15768139 | AGAGGCTGTTTGCAACCATAG | 59.4 | ACCCCTCAAGTTTGATGACG | 60.0 |
| MSB250 | S_15769428 | CATGCAAGCATACACCTATGG | 59.1 | ACAATGTGCCGAGCAGAAG | 60.0 |
| MSB251 | S_16007099 | TCCTGGTCTCAAAAGAGGAAAG | 59.9 | TCCCCATTTGGATTGAAGC | 60.8 |
| MSB252 | S_16103582 | ACGCGTCATTTAAAGCATCC | 60.1 | TGAATTCTGAGAACAGTGAGCTG | 59.7 |
| MSB253 | S_16104457 | GTCTGCCCTACCGACATTG | 59.1 | AAATGGGTGAGGGGTCTAATG | 60.1 |
| MSB254 | S_16421596 | GATCCACGGTCAGGAGACAC | 60.5 | GGAGGGAATAAATGGGAAGC | 59.7 |
| MSB255 | S_16422905 | TCCCTTCCTCTCCTTATCATAATC | 59.0 | CGAGGTTGGTAAATGAGTTGG | 59.5 |
| MSB256 | S_16423170 | TTGGAGGAGTGGTGTTCAGTC | 60.1 | TGTTGTATCTGCTCTTGAAGCTG | 59.7 |
| MSB257 | S_1657199 | ATCTTCGGGCTTCCTTTCC | 60.5 | CGACCTGATGCTCTTGTTCAG | 61.0 |
| MSB258 | S_16573974 | ACACAAGGCCGACAAACTTC | 60.2 | GCTTTGGCATATGGAACTGG | 60.5 |
| MSB259 | S_1659018 | GCTTTTGAAGATCAGAAAAGCTG | 59.7 | GTGACTTCAGGTGCTGTTGG | 59.3 |
| MSB260 | S_16898399 | CAGAAGGGATAAGATCCGACTG | 60.1 | GCAACTCATGAGCTCTCCTTTC | 60.5 |
| MSB261 | S_16898496 | GATGCGTTGCGTATCTAATGTC | 59.6 | CTCGGTAGTGAATCCCATCG | 60.5 |
| MSB262 | S_17402455 | CCGAATGTATATGCCCGAAC | 60.2 | TTACATACCTCTGCTGGAACCAC | 60.4 |
| MSB263 | S_17477999 | GAAAGTGGCTCGGTATGCAC | 60.7 | TCCAAGTATGCGGGATGTTC | 60.9 |
| MSB264 | S_17731289 | GTTGCTAAGAACTGAATCGATGG | 60.1 | CAAAGTACTTGGGATTACGAAAGG | 60.3 |
| MSB265 | S_17762744 | CTGAGCACGCACTCGTAAAG | 59.8 | TCTTTTTGTCTGTGGAAATGTAGC | 59.7 |
| MSB266 | S_17763007 | GGCCATTAAAATTTCTGGACTG | 59.8 | TCGCCGGATGCTGATTAC | 60.8 |
| MSB267 | S_17957691 | CATGTGCTTGAAGCGTTCTC | 59.6 | ATGGACCAGGCTTCACAATC | 59.9 |
| MSB268 | S_18037883 | AGATTTCAACTGAAGATCACATGC | 59.7 | GCCAATAGGAAGAAAAACCTATCC | 60.5 |
| MSB269 | S_18140095 | CTTCGTGACGACATCAAAATG | 59.2 | CCACATGGGTTGGTGTTG | 59.2 |
| MSB270 | S_18140337 | AGGGTGCGTGGAGAAGAAC | 60.3 | ATGCAGACCCCTTTGTTCAG | 60.1 |
| MSB271 | S_18158653 | GCACTGAAAGGCAAAAATCC | 59.7 | TCCTCCTTTGCCATGGTATC | 59.9 |
| MSB272 | S_18160105 | TGGGAAACCTAAAAACATCACC | 60.1 | GCACATGATGAGGAGACTGC | 59.4 |
| MSB273 | S_18374677 | CGCTGCATACTTTTTAATCTCATC | 59.4 | TGAAGGAGTGGTGGCTGATAG | 60.3 |
| MSB274 | S_18375074 | ATTGCCGAGTCAAGTGAACC | 60.1 | GAAGGAATGGGAAACGAACC | 60.7 |
| MSB275 | S_18412180 | TCTGGAAATTTTAAGAAGCAGTCC | 60.1 | CAAAGGAGAGGCAGTGGTTG | 60.8 |
| MSB276 | S_18413444 | GGCTTGAAGTTAAACCATCTGC | 60.1 | GGATCAATACACCTGAGTGCAAC | 60.8 |
| MSB277 | S_18413491 | TGGTTGCACTCAGGTGTATTG | 59.6 | ATTCATCAGCTTCAAGCATCC | 59.3 |
| MSB278 | S_18926685 | TCAACCCTGCAATGGTCAC | 60.5 | CCCTCTAAGCTCCTTCCTCAG | 59.6 |
| MSB279 | S_18927278 | TGCTGTTTTAGGACAGTTTTCAAC | 59.7 | GAAGATGTAGGACTTGGAATACCC | 59.3 |
| MSB280 | S_19215013 | GTGTCTTTGGCTTGCATTTG | 59.3 | CCCATGTTCCCCATTTAGG | 60.0 |
| MSB281 | S_19252438 | GGCAGGGATTTGGTAACTTG | 59.4 | GGGACAAGCATTCCATCTTC | 59.5 |
| MSB282 | S_19253315 | TTGGTTTAGAGTCCTCTGGTTTG | 59.7 | AGGTTCAGGCAGCTCCAAC | 60.4 |
| MSB283 | S_19500904 | CCGTGACTAACAGTGACAATCC | 59.5 | GGAGGCGAGCACTTCAAC | 59.5 |
| MSB284 | S_20137423 | CCTCAAGGTGGAAGGGATG | 60.5 | TCCTTGTTTTAGGATGAAGTACCC | 59.8 |
| MSB285 | S_20137562 | TGGTGGACAATGTCTTCAGC | 59.7 | TTGCTAATGCTCTCCTGACG | 59.2 |
| MSB286 | S_20360438 | AACTCATCGGCTTCAGCAAG | 60.5 | CAAGAAGCAGATTGAGGATGC | 60.0 |
| MSB287 | S_20361140 | TCTGCTTCTTGAACCATCTTCTC | 60.0 | GGCAGAAGAACAAGATTACCATC | 59.2 |
| MSB288 | S_20485495 | GCCTTAGGAACACACCATCC | 59.4 | CAGCAGAATCCACCATCAAC | 59.1 |
| MSB289 | S_20485574 | GTTGATGGTGGATTCTGCTG | 59.1 | CTTCGGCCTTTTCAAAACTG | 59.9 |
| MSB290 | S_21224987 | GGCCAGGAGATACAGTTTTGG | 60.9 | TTCTGATTCTGGCCTGCAC | 59.9 |
| MSB291 | S_21225992 | TGCTTGGGCATAACACAGAC | 59.7 | GTACATTTATAGCCTTGGCAACC | 59.0 |
| MSB292 | S_21424554 | TTCAGAAGCACAAGACAGACAAG | 59.7 | TCCTGTTTTCTAGCCTCAGAGC | 60.2 |
| MSB293 | S_21538834 | CCATTATGTCTCCGTCATCTAAAG | 59.1 | GTTGAGGAGGGTATGGAATTTG | 59.7 |
| MSB294 | S_21719148 | AAATGCAATGCCTGGAGAAG | 60.2 | GTTCCGGATCAAACTATTCAGG | 59.8 |
| MSB295 | S_22031358 | CGGTGTCGATCATAACCTCTC | 59.6 | GGCTCAAAGAGCAAGAGAAGAC | 59.8 |
| MSB296 | S_22278032 | CCCAGAATCCTGCCTTATTTG | 60.8 | TGTCCAACTGGAAACCTTGG | 60.9 |
| MSB297 | S_22425729 | GGCCGCTTCATCATGTTC | 60.2 | GGCTTTAGTAGCATCTTCCTGTG | 59.5 |
| MSB298 | S_22426106 | GGCAGTCCCAGAAGAGGAG | 59.9 | CTGCGATAACCTGGACTGG | 59.3 |
| MSB299 | S_22643242 | CAGCAACTGAATCTGCCAAG | 59.6 | CACTTCCATCTTGTACGACTTTTG | 60.1 |
| MSB300 | S_22643343 | CCAAAAGTCGTACAAGATGGAAG | 60.0 | GTTGAAGATGGCAGGGACTG | 60.7 |
| MSB301 | S_23507105 | GGGCTACTCATCCCAACG | 59.0 | TCAAACATCCACAGCCAAAG | 59.7 |
| MSB302 | S_23507378 | CAGGAGCCTGTACAGAGAATTTG | 60.3 | TCCACATGGATACACCCTCTG | 60.8 |
| MSB303 | S_23889479 | AGGTCCACAAGCCTTTTCC | 59.1 | GTTTCAGGGGTTATGCTTCG | 59.6 |
| MSB304 | S_23889982 | ACCATGGAGATGGTGTGGAG | 60.8 | CGCCAGTACCAGGAAAATAATC | 59.9 |
| MSB305 | S_23943599 | CTCCTAGGAATGCTGACAAGC | 59.1 | GCTCCAAGAACATTTGGACTG | 59.7 |
| MSB306 | S_23944291 | AGCTTGCAAAGGCTCTTGTC | 59.8 | GTTGTGGGCCGGATTCTAC | 60.3 |
| MSB307 | S_24020129 | GTTCCCAATAAAAGTGGACGAC | 59.8 | TGAATGGATTCCCAACAATG | 59.2 |
| MSB308 | S_24021090 | CAGATTGACAGCCAGCTTCC | 60.9 | GAGCGGGGTTACATGTTGTC | 60.4 |
| MSB309 | S_2430993 | GACTTAATGCTTCACAATCTCTCG | 59.5 | AGAGGAGGCTGTGATCTGATTC | 59.9 |
| MSB310 | S_24632710 | GACACCGTGATGTCAGATGC | 60.1 | GGAATATTCTCGATCCCCATC | 59.6 |
| MSB311 | S_24634399 | GATCGGTACCCCATCAGAAG | 59.4 | GACGGCGGTAAGGAGGTAG | 59.7 |
| MSB312 | S_25043941 | GGATCAATACCAGCAAAAACATC | 59.7 | TCGTTTGCTCTTCTCTTCTTTTTC | 60.5 |
| MSB313 | S_25756083 | CATCATCATCGCTGTCAGAAC | 59.3 | CAGCACAACGCAATAGAAGC | 59.6 |
| MSB314 | S_2612672 | GGCATCTCATTTTCATCATTTACC | 60.9 | ATCTTACAAAGAAGTCGCTGCTG | 60.1 |
| MSB315 | S_2619340 | GACTTGGACTCCATCACATGC | 60.5 | AGGAGGAGAGGGTGATCAGAG | 59.8 |
| MSB316 | S_26522514 | AGCTGCCCCAAACAAAGTC | 60.3 | GGTGGATCCATGCTCTTAGC | 59.7 |
| MSB317 | S_26522578 | CAGACATGGCTAAGAGCATGG | 60.8 | GGAGCATTTGAAGAGCTAGGTG | 60.4 |
| MSB318 | S_2880340 | GAGCCATACTTAGCAGCACATC | 59.0 | ACTTGCTTCGGTAAATGGAAAC | 59.5 |
| MSB319 | S_3087867 | CATGTTTCGTCGGGCTTC | 60.2 | GGAGTCACTCGCTCGATCTTAC | 60.4 |
| MSB320 | S_3219932 | GCAGTCGAAGAAGAGAAATTCG | 60.5 | ACAGAAGGCGACTTAGATTTCG | 59.9 |
| MSB321 | S_3221898 | AGTGGACTATGAACAATCTGTTGG | 59.5 | GGCTTGCCAATCAAATCC | 59.0 |
| MSB322 | S_3476724 | TGCTGAGAGTGATTCTGTTGTTG | 60.5 | GCACTTCCTCCATTTGAACC | 59.5 |
| MSB323 | S_3477543 | GGTACCAAATGGTAATGGTAATGC | 60.6 | ACCTCCGTTCAACCTCTGC | 60.3 |
| MSB324 | S_3692365 | GAGCAGAAGCAATCGGAGAC | 60.1 | TGCATGAATGCAAATTCTCAG | 59.8 |
| MSB325 | S_3693194 | GCCGAAATGATGATGGATG | 59.8 | GGACTCGCAAAAGAGACCAG | 60.0 |
| MSB326 | S_3766145 | TGGATGTCCCTTACCTCCAC | 59.8 | TCCCCATAGACCAGATTCTTTG | 60.3 |
| MSB327 | S_4705885 | ATCCACCAGGCATGGAGAC | 60.9 | GCAAGCTTCGAAATCAGGTC | 60.0 |
| MSB328 | S_4740571 | GGCGAGTGCTTGTCCAAC | 60.4 | CTTGCTACAAAAGCCCTTGG | 59.9 |
| MSB329 | S_4821520 | TCATTCGCAGGTCCATCTAAC | 60.1 | TGCTCCTGAATGACTTTTGAAAC | 60.6 |
| MSB330 | S_4821947 | AGAACGCTATGTGAGGACGAG | 59.5 | AATGCCAAACCCCATCATAG | 59.7 |
| MSB331 | S_4822201 | GATTCCCTCTTTTACCTCTTCCAC | 60.7 | CCACCTCCTTAGTAAAGGGAATG | 60.2 |
| MSB332 | S_4956963 | TGTCTGTTCATAGTTTTGGCTTG | 59.3 | AATATGGGCATCAGGCATTC | 59.8 |
| MSB333 | S_500068 | AGCAAGCCATTCTTTGTTGC | 60.4 | GAGATGAGATGCCGCTCTG | 59.6 |
| MSB334 | S_500201 | GCATCTCATCTCCATTGACG | 59.2 | ATTGCCATAATCCCCACAAG | 59.7 |
| MSB335 | S_5161734 | TTCCATCGGTGTGCCTAATC | 60.9 | TTGGCCTTGGTCCATCTG | 60.6 |
| MSB336 | S_5162093 | GATCACACCAATACTCCAGACG | 59.5 | TCCCGAGGTTTTGCATAGAG | 60.2 |
| MSB337 | S_5256486 | CACCGCAGCAACTTTGATAG | 59.5 | ACCGGTCTGAAGCCATCTC | 60.2 |
| MSB338 | S_5331035 | TGATGCGCCTGCAGTAAG | 59.7 | GGACAACATTCAAAGCTGGTG | 60.5 |
| MSB339 | S_596511 | AAAGGCATATCCTATGCAGCAG | 60.6 | TCAACAAGCAAGGCAGCTAAC | 60.6 |
| MSB340 | S_5975578 | CGGTCAGGTCAGGTTGAATC | 60.5 | AGGTCGCAAACGCGATAC | 59.8 |
| MSB341 | S_5976782 | GCCCTCGACACAAACCATAG | 60.5 | GTTTCTCGCCGAGTTACGC | 60.9 |
| MSB342 | S_5976850 | TTCCTAAGTCGCGGAAGTTG | 60.4 | AAAGCTCTAATCAACCACAAACG | 59.7 |
| MSB343 | S_6269876 | GAGAAAGGGTCGTGAGCAAG | 60.0 | GAAATGGACCAGTCAAACACTG | 59.5 |
| MSB344 | S_6271617 | TATTGCAGCCTGCCTCTCTC | 60.7 | CAGCTTCTGACACCGAGAAAC | 60.0 |
| MSB345 | S_627944 | TGTCGGCACTTAGACACAAGAC | 60.4 | AGGCTTATGGCCTCCTTCAC | 60.6 |
| MSB346 | S_6336246 | CCACTTGCATCCAACTACCC | 60.4 | AGGCGGTGAATGAAGAGATG | 60.2 |
| MSB347 | S_6427072 | GAAGGCTTATGATGAGAAATTCG | 59.3 | TGGCTTTAACTGAAGGCTGTC | 59.5 |
| MSB348 | S_6616015 | CAGTCCTTTCAGCCCATATTTC | 60.0 | TGGCAGCTGGTGATACATTC | 59.7 |
| MSB349 | S_6624605 | GCCTCCGAGTATTGTGGATG | 60.5 | CATGGACAACTGCCAAGAAAG | 60.7 |
| MSB350 | S_6857680 | CTCGGCTCCGTAAATTATCTTC | 59.3 | CATTTGAGTGGATGCATGTTG | 60.0 |
| MSB351 | S_7551870 | TCCACCCTTTTCAACTCCTTC | 60.5 | CCCTCTATCCTTGCCCTTTC | 60.0 |
| MSB352 | S_7554899 | GGGCTAAACCAACAGCATTG | 60.5 | CTGGAATTGAAGTCACAACAGG | 59.6 |
| MSB353 | S_7569075 | GCGCGAATAACAATTCCTTC | 59.7 | GACCAAGAGATCGTGGAGATG | 59.7 |
| MSB354 | S_7571268 | TGTCAGCATCATTTCAACTGC | 59.9 | CGGAGGTACACAGCGTCTTC | 60.9 |
| MSB355 | S_7571605 | TCCCATGGCCAATTCTAATC | 59.7 | TGTTGTTGCAGCAGTCGAG | 59.7 |
| MSB356 | S_7625901 | CATTGACGACAATACCAGTTGAAG | 60.8 | ATGCGGTATCACTGACAACAAC | 59.9 |
| MSB357 | S_767516 | GATTTGATGCTGCTTACTGTGG | 59.8 | CTTCTGAAGCTTACCCAACTCC | 59.4 |
| MSB358 | S_7727848 | GGTTCACTGAGACCCTGCTC | 59.8 | GGTTGACTGGTCCATCATCTG | 60.4 |
| MSB359 | S_7775577 | TGATGTCGAACCACCAACTG | 60.6 | CCCAGTCTATGACATGGTCCTC | 60.8 |
| MSB360 | S_7856599 | TCCAATCCGAAATGGAGAAG | 60.0 | CCTTCGGCCTCCTCTACAC | 59.8 |
| MSB361 | S_7856772 | GGAGGCAGAAAAAGATGAGC | 59.0 | TGCTGTATAGGAATGTGAAGTGG | 59.2 |
| MSB362 | S_7856977 | CTGGTGACACCGAAACATTG | 60.0 | TGGCAAAGCCATCAACAAG | 60.8 |
| MSB363 | S_7906423 | ACATTCACTGCTAACTGGGGTAG | 59.6 | TGCCACTGTTCTCATTCTCTTG | 60.4 |
| MSB364 | S_7906859 | TATCAGCGGTGGTTGCTG | 59.4 | CGATGCTGATGTTCACGTTC | 60.3 |
| MSB365 | S_7907815 | ATCACCCCGACAATCACC | 59.1 | GATGCCTCAAGACAGGAGATG | 59.8 |
| MSB366 | S_8034987 | GAACAATGGTTCGAGAAGAAAAAG | 60.5 | GCTCCACTTCAAACTGGACTTC | 60.3 |
| MSB367 | S_8489823 | CCATCCAGTTGATAATGTCCTG | 59.3 | CTTGCACGACCTTTTGTTTG | 59.4 |
| MSB368 | S_8490736 | CGACGAGGGAGAACGAAC | 59.3 | TCAGAGCCTCTCTCATCTTCG | 59.8 |
| MSB369 | S_8604949 | TGCACCCCACCAACACTAC | 60.4 | AGGTCGAGAGGGCTTTGC | 60.5 |
| MSB370 | S_8616069 | CGGTAAAATTGGTGAGTGGAG | 59.5 | GGCAAGTTTCTTGGTGGTG | 59.1 |
| MSB371 | S_8618534 | TGCAAAGCAGAATCAAGGTG | 60.0 | CTTTAATTCCAGCATCAGCAAG | 59.0 |
| MSB372 | S_8763330 | GACGACCCCGTTCTTCATC | 60.5 | CAGTTTCAATCTCTTCACTGTTCG | 60.3 |
| MSB373 | S_8765660 | AACACTTATGTCAGTCCACATTCC | 59.3 | ATGCCTAGACATTAATCCCATTG | 59.3 |
| MSB374 | S_8826045 | TATGAACCTCCAAGGGATGG | 59.7 | TGTGGATTGGGATCAGGAG | 59.4 |
| MSB375 | S_8826467 | TCGAGTCAATGATCCCAATG | 59.5 | ATTCCCAGTCTTGCAATTGAG | 59.2 |
| MSB376 | S_9083677 | CGCCGTTTTACTTGGTTGAG | 60.7 | AGAGAGGCACAATAGAGTTTCCAG | 60.3 |
| MSB377 | S_9083894 | GCAGAGAGAGAGGGGTAAAGG | 59.5 | TCAGAAGCAATGGTGAGTCG | 60.0 |
| MSB378 | S_9084223 | GCCATATGCTTGGGTTCATC | 60.3 | ACCGACTTCTCACTTCCATAGC | 59.8 |
| MSB379 | S_9141167 | CTCAAACCATCGGCTATCTTG | 59.7 | ATTGGTGCTCCGAAGTGG | 59.6 |
| MSB380 | S_9141392 | TGGACAAACAGGTTCCTTCC | 59.9 | GTTTCAGATTCCTCGTGTTTGG | 60.9 |
| MSB381 | S_9141568 | GGCGAGGACTTGACAGTTTC | 59.9 | CACAGTCATTTTCAGAAGAAGAGG | 59.5 |
| MSB382 | S_9240284 | ATCAAATTGGGCTTTTGAGC | 59.2 | TAGCGGGGAAACTAATGTGG | 60.0 |
| MSB383 | S_9688520 | TAATTGTGGTGACCCACTGC | 59.4 | AGAGATGAGTACCCTATTCCAAGC | 59.2 |
| MSB384 | S_9893871 | GGAGGGCCATTTGGAGTC | 60.4 | TGCAAGAATGCCTGATTGAG | 60.0 |
